# Metabolic Serum Profiles for Patients Receiving Allogeneic Stem Cell Transplantation: The Pretransplant Profile Differs for Patients with and without Posttransplant Capillary Leak Syndrome

**DOI:** 10.1155/2015/943430

**Published:** 2015-11-02

**Authors:** Håkon Reikvam, Ida-Sofie Grønningsæter, Aymen Bushra Ahmed, Kimberley Hatfield, Øystein Bruserud

**Affiliations:** ^1^Section Hematology, Department of Clinical Science, University of Bergen, 5021 Bergen, Norway; ^2^Section Hematology, Department of Medicine, Haukeland University Hospital, 5021 Bergen, Norway

## Abstract

Allogeneic stem cell transplantation is commonly used in the treatment of younger patients with severe hematological diseases, and endothelial cells seem to be important for the development of several posttransplant complications. Capillary leak syndrome is a common early posttransplant complication where endothelial cell dysfunction probably contributes to the pathogenesis. In the present study we investigated whether the pretreatment serum metabolic profile reflects a risk of posttransplant capillary leak syndrome. We investigated the pretransplant serum levels of 766 metabolites for 80 consecutive allotransplant recipients. Patients with later capillary leak syndrome showed increased pretherapy levels of metabolites associated with endothelial dysfunction (homocitrulline, adenosine) altered renal regulation of fluid and/or electrolyte balance (betaine, methoxytyramine, and taurine) and altered vascular function (cytidine, adenosine, and methoxytyramine). Additional bioinformatical analyses showed that capillary leak syndrome was also associated with altered purine/pyrimidine metabolism (i.e., metabolites involved in vascular regulation and endothelial functions), aminoglycosylation (possibly important for endothelial cell functions), and eicosanoid metabolism (also involved in vascular regulation). Our observations are consistent with the hypothesis that the pretransplant metabolic status can be a marker for posttransplant abnormal fluid and/or electrolyte balance.

## 1. Introduction

Allogeneic hematopoietic stem cell transplantation (allo-SCT) is used in the treatment of severe hematological diseases, especially high-risk malignancies [1, 2]. The treatment is associated with a relatively high risk of transplant-related morbidity and mortality due to severe posttransplant complications. Endothelial cells seem to be involved in the pathogenesis of several of these complications, that is, venoocclusive disease, acute graft versus host disease (GVHD), capillary leak syndrome, and microangiopathic hemolysis [3–5]. Furthermore, several studies have demonstrated that pretransplant clinical factors are important for the risk of posttransplant complications; this is true both for organ toxicity and immune-mediated complications and these factors include both disease stage and previous treatment, type of conditioning therapy, and comorbidity (i.e., additional complicating diseases) [6, 7]. Recent studies suggest that even preconditioning factors are important at least for the development of immune-mediated posttransplant complications [8, 9].

The hypothesis that endothelial cells are involved in the development of posttransplant complications is largely based on the observations of altered levels of endothelial markers either prior to [9], or during the development of the complications [3–5, 10–13]. The molecular mechanisms causing altered endothelial cell functions in allotransplant recipients are largely unknown. However, metabolic factors may be important for regulation of endothelial cell functions or the fluid/electrolyte balance, and we therefore investigated whether the preconditioning/pretransplant metabolic serum profile showed any associations with the later development of capillary leak syndrome. This is a relatively frequent posttransplant complication where endothelial, vascular, or renal dysfunctions are likely to contribute.

## 2. Material and Methods

### 2.1. Patients

The study was approved by the local Ethics Committee (Regional Ethics Committee III, University of Bergen, Norway) and samples were collected after written informed consent. The study included 80 consecutively allotransplanted adult patients (51 men and 29 women; median age 44 years with range 16–69 years) from a defined geographic area (Norwegian Health Regions III, IV, and V) and transplanted with a family donor. The decision to perform an allotransplantation was taken by the Norwegian Advisory Board for Stem Cell Transplantation and based on national guidelines. Thus, our study is population-based and includes an unselected and consecutive group of well-characterized patients.

All samples were collected before start of conditioning therapy (median 19 days before). The clinical characteristics of the patient are given in Table 1. Most patients received GVHD prophylaxis with cyclosporine A and methotrexate, and only two patients received cyclosporine A alone. Patients were transplanted with granulocyte colony-stimulating factor (G-CSF) mobilized peripheral blood stem cells derived from HLA-matched family donors (aplastic anemia patients received bone marrow grafts). Neutrophil reconstitution was defined as three consecutive days with neutrophil counts of at least 0.2 × 10^9^/L and platelet reconstitution as stable platelet counts exceeding 20 × 10^9^/L for at least 3 consecutive days. All patients were carefully examined for and classified with regard to comorbidity according to Sorror [7]; none of the patients had liver or renal disease and the overall comorbidity score was low (1 or 0). Sinusoidal obstruction syndrome was not diagnosed in any patient.

### 2.2. Diagnosis of Capillary Leak Syndrome

Capillary leak syndrome was defined as at least 10% weight gain during 24 hours despite diuretic therapy. The patients' weight was registered twice daily, and according to the department's guidelines patients then received 20–40 mg of furosemide if the weight gain exceeded 2.0 kg compared with the body weight registered immediately before start of the conditioning therapy. A clinical evaluation was in addition done twice daily by one of the physicians from the allotransplantation team, and it was then decided whether additional diuretic treatment should be administered. The intravenous fluid administration during conditioning therapy and following stem cell transplantation was administered to maintain a relatively high diuresis (i.e., the amount of intravenous fluid administration was decided based on a volume parameter independent of the patients' body weight), and for this reason we used a definition of capillary leak syndrome based on the absolute and not the relative weight increase.

### 2.3. Preparation of Serum Samples

All venous blood samples were collected into sterile plastic tubes (BD Vacutainer SST Serum Separation Tubes, Becton-Dickenson; Franklin Lakes, NJ, USA) and allowed to coagulate for 120 minutes at room temperature (18°C) before centrifugation (300 g for 10 minutes) and serum collection. All samples were immediately frozen at −70°C until being analyzed.

### 2.4. Analysis of Metabolite Serum Levels

Metabolomic profiling analysis of all samples was carried out in collaboration with Metabolon [14]. Each sample was accessioned into the Metabolon LIMS system and was assigned by the LIMS a unique identifier used to track all sample handling and results. The samples (and all derived aliquots) were tracked by the LIMS system. Samples were prepared using the automated MicroLab STAR system (Hamilton Company, Bonaduz, Switzerland). A recovery standard was added prior to the first step in the extraction process for quality control purposes. To remove protein, to dissociate small molecules bound to protein or trapped in the precipitated protein matrix, and to recover chemically diverse metabolites, proteins were precipitated with methanol under vigorous shaking for 2 minutes followed by centrifugation. The resulting extract was divided into four fractions: one for analysis by UPLC-MS/MS with positive ion mode electrospray ionization, one for analysis by UPLC-MS/MS with negative ion mode electrospray ionization, one for analysis by GC-MS, and one sample that was reserved for backup. Samples were placed briefly on a Zymark TurboVap (KcKinley Scientific, Sparta, NJ, USA) to remove the organic solvent. For LC, the samples were stored overnight under nitrogen before preparation for analysis. For GC, each sample was dried under vacuum overnight before preparation for analysis. A total of 766 metabolites were analyzed in all samples. These metabolites could be divided into the eight main categories (corresponding number of metabolites): amino acids (156), peptides (92), carbohydrates (24), energy metabolism (9), lipids (298), nucleotides (36), cofactors/vitamins (27), and xenobiotics (124).

### 2.5. Analysis of Serum Cytokine Levels

Cytokine levels were determined by Luminex analyses (R&D Systems; Abingdon, UK) and included the following: (i) the immunomodulatory Interferon-*γ* (IFN-*γ*), CD40 ligand (CD40L), and tumor necrosis factor-*α* (TNF-*α*); (ii) the interleukins IL-1*α*, IL1-*β*, IL-1RA, IL-2, IL-4, IL-5, IL-6, IL-8/CXCL8, IL-10, IL-12, IL-13, and IL-17; (iii) the chemokines CCL3, CCL4, CCL5, CCL11, CXCL5, CXCL10, and CXCL11; and (iv) the growth factors basic fibroblast growth factor (bFGF), granulocyte macrophage colony-stimulating factor (GM-SCF), G-SCF, vascular endothelial growth factor (VEGF), thrombopoietin (Tpo), epithelial growth factor (EGF), hepatocyte growth factor (HGF), and Leptin. All analyses were performed in duplicates strictly according to the manufacturer's instructions.

### 2.6. Bioinformatical and Statistical Analyses

Bioinformatical analyses were performed using the J-Express (MolMine AS, Bergen, Norway) [15]. For hierarchical clustering all values were median variance standardized and log⁡(2) transformed. The complete linkage was used as linkage method, and for distance measure the Euclidean correlation was used. Statistical analyses were performed using the Statistical Package for the Social Sciences (SPSS) version 15.0 (SPSS Inc., Chicago, IL, USA). The Chi-Square test was used to compare different groups. Unless otherwise stated* p* values < 0.05 were regarded as statistically significant.

## 3. Results and Discussion

### 3.1. Capillary Leak Syndrome Is a Frequent Complication after Allogeneic Stem Cell Transplantation

Capillary leak syndrome characterized by a weight increase ≥ 5 kg/24 hours was diagnosed in 41 patients; patients with and without the syndrome did not differ with regard to age, gender, body mass index, later acute GVHD, or hematological reconstitution (Table 1). They did not differ with regard to pretransplant hemoglobin level, peripheral blood white blood cell counts, serum CRP, or serum LDH levels either. However, patients with later capillary leak syndrome showed lower peripheral blood platelet counts (see Table 1). According to the definition patients with capillary leak syndrome showed a larger weight gain than the other patients, but in addition their maximal weight increase occurred later than for the other patients (median 1 day before transplant versus 9 days after transplant).

### 3.2. Patients with Posttransplant Capillary Leak Syndrome Show Increased Pretransplant Levels of Several Metabolites Known to Regulate Endothelial Function, Renal Function, or Vascular Permeability

**Figure 1:**
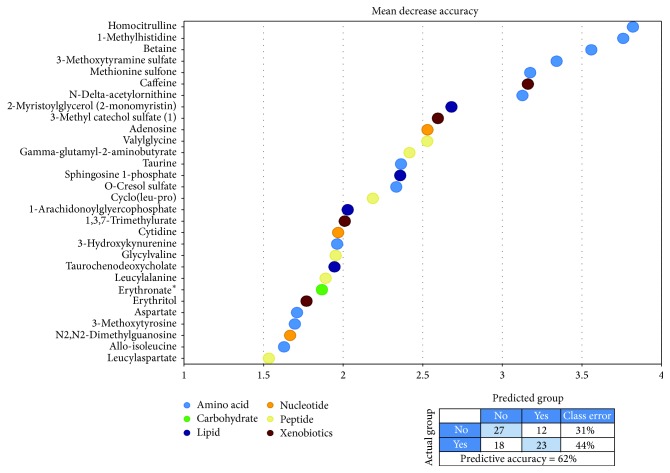
Random forest analysis of pretransplant metabolite levels: identification of metabolites showing altered serum levels in patient with capillary leak syndrome. Random forest analysis could distinguish between the metabolic signatures of patients with and without capillary leak syndrome with a predictive accuracy of 62%, suggesting that these metabolites are candidate biomarkers for increased risk of capillary leak syndrome. The figure presents the top 30 metabolites based on their importance to separate the two patient groups. The different colors reflect the metabolite classification as indicated at the bottom of the figure.

We performed a random forest analysis (i.e., a supervised classification statistical analysis) to identify metabolites that are candidate biomarkers for predicting risk of capillary leak syndrome. We could then identify metabolic biomarkers that distinguished between patients with and without capillary leak syndrome with a predictive accuracy of 62% (Figure 1). This number is greater than the random chance alone (~50%), suggesting that the identified metabolites are candidate biomarkers for predicting risk of capillary leak syndrome. The figure shows the top 30 metabolites ranked according to their importance for separation of the two groups. A majority of these metabolites (12 of all the 30, 6 of the 7 highest ranked) are involved in amino acid metabolism.

The biological characteristics of the 7 metabolites showing strongest association with development of capillary leak syndrome (Figure 1) are summarized in Table 2 [16–32]. Firstly, homocitrulline and ornithine are linked together in the urea cycle [16], but homocitrulline is also a product derived from carbamylation, that is, a nonenzymatic posttranslational protein modification with binding of isocyanic acid to amino groups. Serum homocitrulline levels may therefore reflect the overall carbamylation activity including carbamylation of tissue proteins [17]. Both the serum levels and protein carbamylation seem to be important in vascular biology [17] and have been associated with risk of cardiovascular death [18] as well as increased mortality in renal failure [19] and endothelial dysfunction through carbamylation of low density lipoprotein [20]. Secondly, methoxytyramine is the O-methylated metabolite of dopamine [23] and is therefore used as a marker for dopamine-producing tumors [24], but dopamine is also involved in the renal regulation of body fluid and electrolyte balance [25, 26] and regulation of vascular permeability [27]. Betaine also seems to have a role in vascular biology/fluid balance and adaptation to hypertonic stress [22]. Thirdly, 1-methylhistidine is derived from metabolism of anserine and possibly reflects the nutritional status [21]; the same may be true for methionine sulfone [28, 29] and N^*δ*^-acetylornithine [32]. Finally, serum caffeine levels are not only determined by oral intake but also by several other factors including physical activity, the fat mass, and carbohydrate intake (i.e., nutritional status) [30, 31]. To conclude, several metabolites showing strong associations with fluid retention may influence endothelial cell function, modulate renal regulation of fluid/electrolyte homeostasis, or reflect the nutritional balance.

### 3.3. The Pretransplant Metabolite Profile Can Be Used to Distinguish between Patients with and without Posttransplant Capillary Leak Syndrome

**Figure 2:**
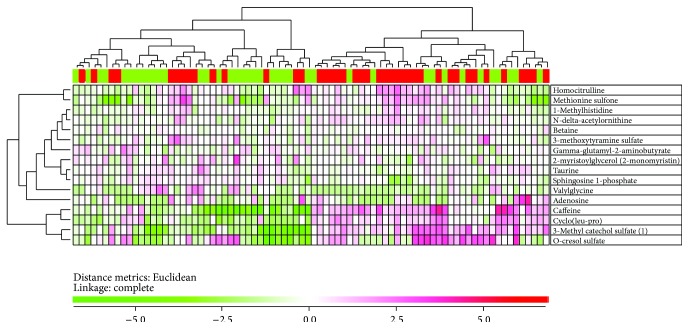
The metabolic profile can be used to distinguish patient with and without capillary leak syndrome. We performed an unsupervised hierarchical clustering analysis (Euclidean distance measure with complete linkage) based on the 15 highest ranked metabolites from the random forest analysis (Figure 1). The figure demonstrates the heat map with corresponding denograms. The patient classification is shown at the top; those patients with capillary leak syndrome and weight increase ≥5 kg are marked with red and the others are marked with green. The left main cluster included a majority of patients without capillary leak syndrome (14 out of 40 patients) whereas a majority of the patients in the right main cluster developed the syndrome (27 out of 40 patients; Chi-square test,* p* = 0.0036).

We performed an unsupervised hierarchical clustering analysis (Figure 2) based on the 15 highest ranked metabolites from the random forest analysis (Figure 1). The upper main cluster included most of the amino acid metabolites whereas the lower included caffeine, cyclo (leu-pro), 3-methyl catechol sulfate, and o-cresol sulfate. The patients formed two main clusters with 40 patients each. The left cluster included relatively few patients with capillary leak syndrome (14 out of 40 patients) and the right cluster included a majority of patients with capillary leak syndrome (27 out of 40 patients); this difference in frequency of patients with capillary leak syndrome reached statistical significance (Chi-square test,* p* = 0.0036).

### 3.4. Patients with Capillary Leak Syndrome Show Altered Pyrimidine and Purine Metabolism: Possible Effects on Endothelial Cells and Vascular Smooth Muscle Cells

**Figure 3:**
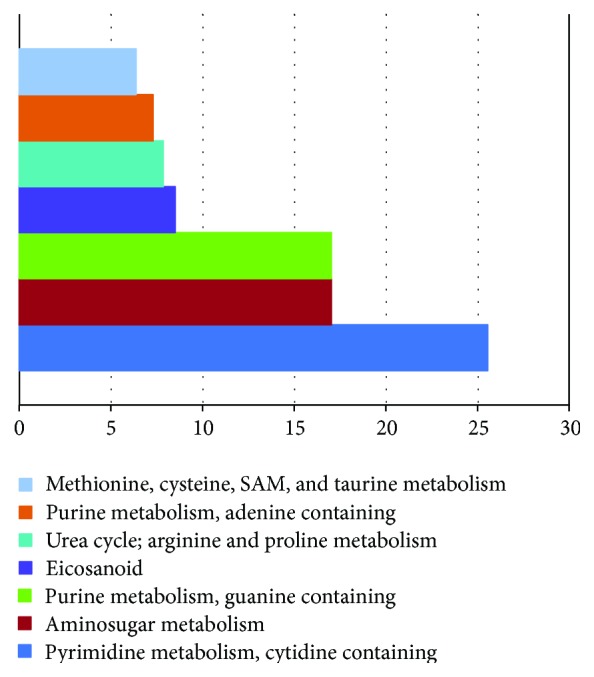
Pathway enrichment analysis of metabolic profiles associated with capillary leak syndrome. The pathway enrichment analysis was used to identify metabolites/pathways that were altered in pretransplant samples derived from patients with capillary leak syndrome (i.e., weight increase >5 kg) compared to patients with lower weight increase (i.e., <5 kg). A pathway enrichment value >1 indicates that the pathway was increased in patients with an acute phase reaction. The top ranked metabolic pathways (*p* < 0.01, enrichment value >5) identified by this comparison are given in the figure.

We did a pathway enrichment analysis to compare patients with and without capillary leak syndrome. Several metabolic subclasses/subpathways were enriched among capillary leak patients; the pathways showing an enrichment value >5 (*p* < 0.01) are presented in Figure 3 and an overview of their significantly altered metabolites is presented in Table 3 [33–42]. A hierarchical clustering analysis based on all the 53 metabolites belonging to the first ten ranked metabolic pathways (Figure 3) could not distinguish between patients with and without capillary leak syndrome (data not shown). The term “Pyrimidine metabolism, cytidine containing” (enrichment value 25.53) got the highest score in this analysis and “Purine metabolism guanine containing” got the second highest (17.02). Purinergic signaling is important for regulation of vascular tone and there is a metabolic interaction between guanine and adenine; ATP then seems to have a dual function as it can be released as a neurotransmitter to cause vasoconstriction or be released by endothelial cells as a stress response leading to vasodilation [35].

Nucleotides bind to several P2X and P2Y receptors that are expressed both by endothelial cells (e.g., P2Y_1_, P2Y_2_, P2Y_4_, and P2Y_6_ that bind ATP, ADP, UDP, and UTP) and vascular smooth muscle cells [35]. Endothelial cells are thus important for the vascular purine effects because they express these receptors and thereby are targets for nucleotides released from myocytes and blood cells but also because they are a source of purines and contribute to purine metabolism through expression of cell surface ectonucleotidases that regulate local nucleotide levels [35]. The dominant receptors on endothelial cells are A_2A_ and A_2B_ adenosine receptors and P2Y_1_, P2Y_2_, and P2X_4_ nucleotide receptors [43].

### 3.5. Capillary Leak Syndrome Is Associated with Altered Pretransplant Aminoglycosylation: Relevance for Vascular Glycocalyx, Glycosylation of Endothelial Surface Molecules, and Vascular Permeability

The endothelial cell barrier is regulated by cell-cell and cell-extracellular matrix adhesion. The glycocalyx is an extracellular covering on the luminal side of the endothelium, and it is an important regulator of endothelial permeability together with the transcellular and paracellular pathways described below [44]. This surface layer consists of proteoglycans, glycosaminoglycans, and adsorbed plasma proteins; its thickness as well as its negative charging varies between vascular beds/tissues [44]. This barrier functions as a sieve allowing transendothelial transport of molecules but inhibiting cellular extravasation. Several experimental studies suggest that the degree of glycosylation of the surface layer molecules is an important regulator of vessel permeability; and the permeability can be altered through hydrolysis of sialic acid moieties, neuraminidase treatment, and degradation of heparan sulphate [44].

Endothelial cells express several adhesion molecules that are important for leukocyte trafficking and these molecules can be N-glycosylated; this is true for ICAM-1, P-selectin, and E-selectin [45]. The carbohydrate profile of the endothelial surface differs between vascular beds and glycosylation is a dynamic process that is further modified during inflammation. Thus, glycosylation is an important regulator of vessel/endothelial permeability both through modulation of the surface glycocalyx and the luminal endothelial cell surface molecules.

The mechanisms for regulation of endothelial permeability were reviewed recently [46]. The endothelial permeability for molecules from 0.1 nm (sodium ions) up to 3.6 nm shows an inverse correlation with molecular size, whereas the permeability seems independent of the molecular diameter for larger molecules [44, 47]. These differences correspond to two different transport mechanisms. The transport of the larger molecules (e.g., immunoglobulin IgG, albumin) occurs through a transcellular mechanism referred to as transcytosis or vesicular transport, whereas the smaller molecules (i.e., water and ions) can pass through interendothelial junctions in a paracellular pathway [46]. The regulation of both these pathways is important to maintain the endothelial barrier. The paracellular pathway is regulated by several junctional proteins: (i) tight junctions are formed by occludins, claudins, and junctional adhesion molecules (JAM) and especially JAM-B is detected in endothelial cells; (ii) GAP junctions are formed by connexins; and (iii) adherent junctions represent homotypic adhesion between cells and are formed by vascular endothelial (VE) cadherins. Tight junctions and adherent junction are linked to the intracellular cytoskeleton, whereas the GAP junctions represent channels between cells that allow molecular exchange of signaling molecules and ions. As can be seen from Table 4 the function of several molecules involved in the formation of all these permeability-regulating structures can be modulated by glycosylation [48–60].

### 3.6. Increased Pretransplant Thromboxane B_2_: Is Altered Eicosanoid Metabolism Important in the Development of Capillary Leak Syndrome?

Thromboxane B_2_ was the only mediator in this class that was significantly altered (Table 3). This mediator is the main degradation product of Thromboxane A_2_ [36] that can be released by endothelial cells and bind to specific receptors expressed both by endothelium and vascular smooth muscle cells [36–38]. This receptor ligation/activation usually promotes vasoconstriction [36].

### 3.7. Pretransplant Cytokine Profiles Reveal Patients with Increased Risk for Capillary Leak Syndrome

Serum cytokine profiles were available for the first 56 out of our 80 patients. We first compared the serum levels of each cytokine for patients with and without capillary leak syndrome. CD40L was then the only mediator that differed significantly between the two groups (*p* = 0.0061, Mann-Whitney *U* test), and these levels were lower in patients with capillary leak syndrome. However, CD40L is released by platelets, and patients with capillary leak syndrome showed decreased pretransplant platelet counts (Table 1) and a strong correlation between platelet count and serum CD40L levels. The most likely explanation for the difference in CD40L serum levels is thus a difference in CD40L release by platelets during* ex vivo* handling of the samples [61]. Although the CD40/CD40L system is expressed both by endothelial cells and vascular smooth muscle cells [62] and thus may be important in the development of capillary leak syndrome [63, 64], the use of serum samples in our present study makes it impossible to evaluate whether CD40/CD40L is important for the pretransplant predisposition to capillary leak. A possible explanation for the association between decreased pretransplant platelet counts and posttransplant capillary leak syndrome could be a more severe cell damage caused by previous chemotherapy causing both endothelial cell damage and delayed hematopoietic reconstitution.

**Figure 4:**
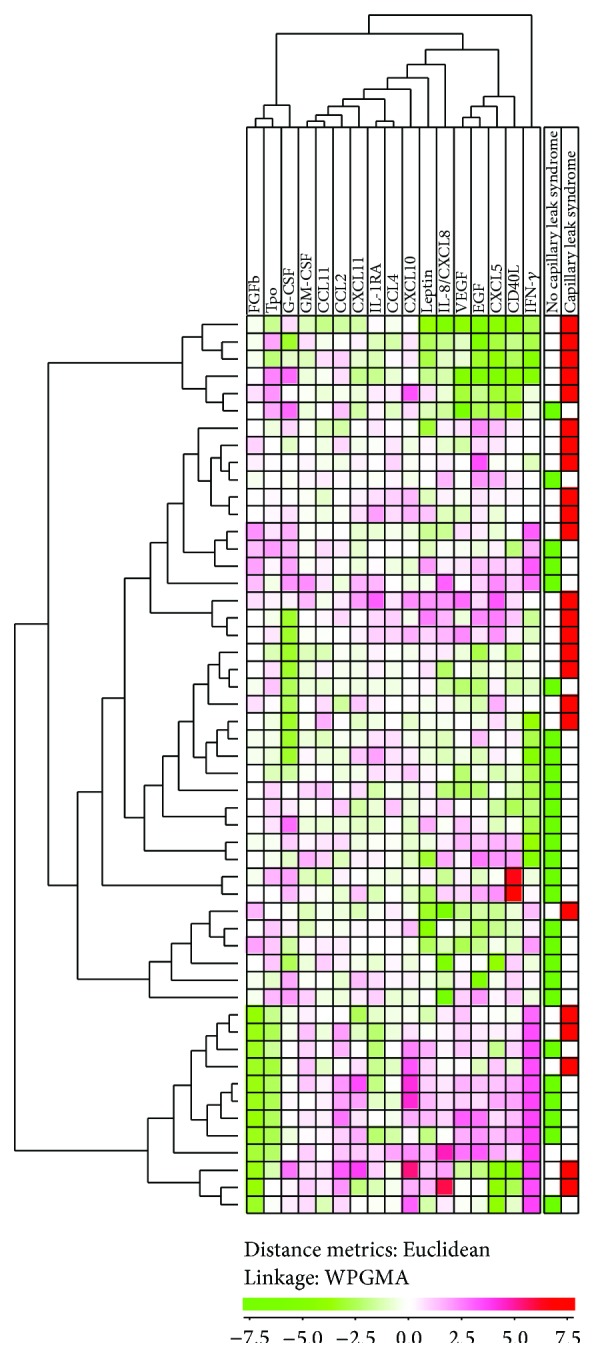
Hierarchical clustering based on the cytokine profiles: a study of the first 56 consecutive patients. Based on the pretransplant cytokine levels we performed an unsupervised hierarchal clustering analysis (Euclidean distance measure with WPGMA linkage). The figure presents the heat map with corresponding dendrograms. The horizontal cytokine clustering is seen at the top of the figure and the vertical patient clustering at the left part of the figure. Red color indicates high levels and green color low levels. The right column shows the distribution of patients with capillary leak syndrome, that is, weight increase ≥5 kg marked with red bars and the others marked with green.

Furthermore, we also performed unsupervised hierarchal clustering analyses based on the pretransplant cytokine levels. Cytokines that were detected only in a minority of patients (Il-1*α*, IL-1*β*, IL-2, IL-4, IL-5, IL-6, IL-10, IL-17, IL-22, CCL3, and TNF-*α*) were left out from the bioinformatical analysis (Figure 4). The frequency of patients with capillary leak syndrome did not differ significantly between the two main clusters. The majority of patients later developed capillary leak syndrome clustered in the upper part, that is, 18 of the upper 24 patients (Figure 4). In contrast only six of the lower 32 patients developed capillary leak syndrome (Figure 4). This observation suggests that many patients with capillary leak syndrome show similarities in their pretransplant cytokine profile, but in contrast to the metabolite clustering (Figure 2) these similarities are not sufficient to cause statistically significant differences between main clusters. Thus, our hypothesis is that metabolic differences are more important than cytokine differences for the predisposition to capillary leak syndrome in allotransplant recipients.

## 4. Conclusions

In our present study we describe an association between the pretreatment/pretransplant metabolic profile and development of fluid retention/capillary leak syndrome after allogeneic stem cell transplantation. This pretransplant metabolic profile may thus represent a predisposition to posttransplant fluid retention. Bioinformatical analyses suggest that this association is caused especially by altered purine and pyrimidine metabolism as well as aminoglycosidation and eicosanoid metabolism. Several metabolites that show different levels are known to induce endothelial dysfunction or altered vascular and renal functions. We have previously described that pretransplant factors reflect the risk of acute GVHD after allotransplantation [8, 65], and our present observations suggest that pretransplant metabolic factors are important also for the risk of posttransplant fluid retention.

## Supplementary Material

The description of the Supplementary Material should read: Supplementary Table 1. Classification of all metabolies analysed in the study, the pathways showing pretransplant alterations in patients with posttransplant capillary leak syndrome are marked with yellow. The marked pathways represent an extended list compared with the pathways presented in Figure 3; the ratio from the bioinformatical analysis is given for each Sub Pathway in parenthesis.

## Figures and Tables

**Table 1:** Patients with and without capillary leak syndrome: a comparison of clinical characteristics.

	Weight increase			
	All patients	<5 kg (*n* = 39)	≥5 kg (*n* = 41)	*p* value
Demographic data				
Age (years)	44 (15–69)	42 (15–69)	51 (20–67)	0.1066
Gender (female/male)	29/51	13/26	16/25	0.5966
Weight (kg)	71.4 (41.5–110)	73 (41.5–110)	70 (46.5–133)	0.7031
Height (cm)	176 (149–197)	179 (149–193)	174 (158–197)	0.4102
BMI (kg/m^2^)	23.2 (16.6–39.7)	23.5 (16.6–36.5)	23.1 (17.9–39.7)	0.2852

Diagnosis				
AML	35	19	16	
MDS	15	5	10	
ALL	18	11	7	
CML	4	1	3	
CMML	2	0	2	
CLL	1	1	0	
PMF	2	1	1	
AA	3	0	3	

Body weight				
Weight increase (kg)	5.0 (0–16.1)	3.2 (0–4.8)	7.0 (5.0–16.1)	**<0.0001**
Day of maximal weight	6 (−8–44)	−1 (−8–13)	9 (−5–44)	**0.0011**

Acute GVHD (yes/no)	42/28	24/12	18/16	0.2414

Reconstitution				
Neutrophil (day post-SCT)	15 (6–52)	15 (6–29)	16 (11–52)	0.2848
Platelets (day post-SCT)	15 (9–33)	14 (9–29)	16 (9–33)	0.2076

Baseline pretransplant status				
Leukocytes (×10^9^/L)	3.9 (0.5–44.3)	3.5 (0.7–11.8)	4.8 (0.5–44.3)	0.1972
Hb (g/dL)	10.5 (7.8–14.1)	10.6 (7.8–14.0)	10.4 (7.9–14.1)	0.4440
Platelets (×10^9^/L)	143 (10–721)	205 (10–721)	119 (10–554)	**0.0147**
CRP (mg/L)	5 (1–120)	5 (1–64)	6 (1–120)	0.1660
LDH (IU/dL)	185 (92–1665)	190 (129–489)	174 (92–1665)	0.1780

Unless otherwise stated values are given as median (variation range). Height and weight were registered at the start of conditioning therapy. For statistical analysis the Mann-Whitney test was used to compare continues variables and the Chi-square test for categorical variables.

AA, aplastic anemia; ALL; acute lymphoblastic leukemia; AML, acute myelogenous leukemia; BMI, body mass index; MDS, myelodysplastic syndrome; CLL, chronic lymphocytic leukemia; CML, chronic myelogenous leukemia; CMML, chronic myelomonocytic leukemia; CRP, C-reactive protein; GVHD, graft versus host disease; Hb, hemoglobin; LDH, lactate dehydrogenase; PMF, primary myelofibrosis; WBC, white blood cell count.

**Table 2:** The biological functions of the seven metabolites showing the largest difference when comparing pretransplant serum levels for patients with and without posttransplant capillary leak syndrome (random forest analysis, see Figure 1): a summary of known effects on endothelial cells, renal function, and vascular permeability.

Metabolite (main class)	Biological functions relevant for fluid and electrolyte balance
Homocitrulline (amino acid)	Homocitrulline and ornithine are linked together in the urea cycle, and genetic defects in ornithine transport into mitochondria cause increased systemic homocitrulline levels [16]. Homocitrulline is also a product derived from carbamylation, a nonenzymatic posttranslational protein modification with binding of isocyanic acid to *ε*-amino groups of lysine, and serum/plasma homocitrulline levels may reflect the overall carbamylation process including carbamylation of tissue proteins [17]. Serum homocitrulline levels and carbamylation seem important for vascular biology, and high plasma citrulline is associated with severe coronary artery disease [17], risk of cardiovascular death [18], and increased mortality in renal failure [19]. It is not known whether homocitrulline/carbamylation is important for regulation of vascular permeability or regulation of paracellular or transendothelial transport, but carbamylation of low density lipoprotein induces endothelial cell dysfunction [20].

1-Methylhistidine (amino acid)	Anserine (beta-alanyl-1-methyl-L-histidine) is present in many kinds of vertebrate muscles but not in human muscles; 1-methylhistidine is derived from metabolism of anserine and may thus reflect the nutritional status of the patients [21].

Betaine (amino acid)	Betaine is found in many foods including spinach and wheat, and it accumulates in renal medullary cells during adaptation to hypertonic stress [22]. The primary role of betaine in the kidney seems to be osmoprotection; intracellular accumulation is then mediated by the betaine/GABA transporter. Thus, betaine seems to be involved in renal regulation of fluid balance.

Methoxytyramine (amino acid)	This is the O-methylated metabolite of dopamine [23] and recent studies suggest that it can be used as a marker for dopamine-producing tumors [24]. Dopamine is involved in renal regulation of body fluid and electrolyte balance; these effects are mediated through binding to specific dopamine receptors that regulate the function of Na^+^/K^+^-ATPase [25]. Animal studies suggest that altered dopamine-induced signaling is important for fluid retention in nephrotic syndrome [26]. Thus, the increased methoxytyramine levels may reflect altered dopamine metabolism that contributes to fluid retention through a renal mechanism. Dopamine may also be important for fluid extravasation in other vascular beds [27].

Methionine sulfone (amino acid)	Methionine can be oxidized to methionine sulfone during food processing; this metabolite seems to reduce the effectiveness of gut proteases to digest dietary proteins and its plasma/serum levels may reflect the nutritional status [28, 29].

Caffeine (xenobiotics)	Serum caffeine levels are not only determined by the intake but are rather determined by several additional factors, including physical activity, the fat mass, and carbohydrate intake (i.e., nutritional status) [30, 31].

N^*δ*^-Acetylornithine (amino acid)	This is a nonprotein amino acid found in various plants [32]; its level may thus be related to the nutritional status.

**Table 3:** The table shows the following: (I) the main metabolic classification (referred to as Super Pathway in the Supplementary Table in Supplementary Material available online at http://dx.doi.org/10.1155/2015/943430) together with the number of significantly altered metabolites belonging to this class relative to the total number of examined metabolites from this class; (II) the subclass(es) (subpathway, see also Figure 3) within the corresponding main class showing an enrichment value >5 followed by the number of significantly altered single metabolites relative to the total number of investigated metabolites for this subclass; and (III) the single metabolites showing significant differences, whether they were increased (↑) or decreased (↓) for patients with capillary leak syndrome, and a brief summary of known and relevant functional effects on endothelial cells, renal function, or vascular biology. The main class/subclasses are ranked according to the score presented in Figure 3 (value given in parenthesis after the subclass identification). The nucleotide main class (super pathway) included three different subclasses/subpathways that showed an enrichment value >5; 6 of the 36 metabolites in the nucleotide class differed significantly between the two patient subsets (2 in each subclass, indicated as 6/36). The corresponding numbers for the Main Class Amino acids were 7/171, 2, and 5, respectively, for 2 different subclasses.

(I) Main metabolic classification (super pathway)	(II) Subclass (subpathway)	(III) Metabolite (biochemical name)
Nucleotide 6/36	Pyrimidine metabolism, cytidine containing 2/2 (25.53)	↑*cytidine*, ↑*N4-acetylcytidine* Animal models suggest that cytidine can function as a cardiovascular regulator through binding to adenosine A1 and A2 receptor [33].

Carbohydrate 2/24	Aminosugar metabolism 2/3 (17.02)	↑*glucuronate* Glucuronate is important for the synthesis of several glycoproteins (see Table 4). ↑*erythronate* Erythronic acid is important in mitochondrial metabolism, and it is normally present only at low levels [34].

Nucleotide 6/36	Purine metabolism, guanine containing 2/6 (17.02)	↑*7-methylguanine*, ↑*N2,N2-dimethylguanosine* There is a metabolic interaction between adenosine and guanosine metabolism; extracellular ADT and ATP are known angioregulatory mediators [35].

Lipid 1/303	Eicosanoid 1/6 (8.51)	↑*Thromboxane B2* Thromboxane B2 is the main degradation product of Thromboxane A2 [36] that can be released by endothelial cells and bind to specific receptors expressed both by endothelium and smooth muscle vascular cells [36–38]. Receptor activation usually promotes vasoconstriction [36].

Amino acids 2/172 (7.86)	Urea cycle; Arginine and Proline Metabolism 2/13	↑*homocitrulline*; ↑*N-delta-acetylornithine* Both metabolites are important for the urea cycle; ornithine enters mitochondria and reacts with carbamoyl phosphate to form citrulline that enters the urea cycle.

Nucleotide 6/36	Purine metabolism, adenine containing 2/7 (7.30)	↑*adenosine*, ↑*N6-carbamoylthreonyladenosine* Adenosine is a well-characterized cardiovascular regulator and binds to specific receptors [39, 40]. A2A and A2B receptors are also expressed by endothelial cells [66].

Amino acids 5/172	Methionine, cysteine, SAM, and taurine metabolism 5/16 (6.38)	↑*N-acetylmethionine*; ↑*N-formylmethionine*; ↑*Methionine sulfone* N-acetylmethionine can be formed by normal cells from methionine, but it is also a bioavailable form of methionine in humans [41]. Methionine sulfone may reflect the nutritional status. ↓*hypotaurine*; ↓*taurine* As reviewed recently taurine is a *β*-amino acid that is not incorporated into proteins and can serve as an intracellular osmolyte [42]. It is one of the major osmolytes (together with betaine) in the renal medulla. A renal adaptive response to dietary intake seems to conserve the total taurine body pool through the balance between reabsorption and excretion. Taurine seems to be a regulator of renal blood flow and influences the flow within all types of vessels, and it also seems to stabilize the endothelium.

**Table 4:** The importance of protein glycosylation for paracellular and transcellular transport across the endothelial cell layer.

Cellular structure and molecule	Glycosylation	References
*Tight junctions*		
Claudin-1, -2, and -4	Two in silico studies suggest that claudins can be glycosylated, and there seems to be a functional interplay between glycosylation and phosphorylation. For claudin-1 it has been suggested that alternate phosphorylation/glycosylation on Ser192, Ser205, Ser206, and Thr191 may provide an on/off switch to regulate their assembly at tight junctions.	[48, 49]
Occludins	An in silico study suggests that human occludin can be O-*β*-glycosylated, and for Ser408 and Ser490 there may be a functional interplay between phosphorylation and glycosylation. The glycosylation can be altered by cellular stress.	[50, 51]

*Gap junctions*		
Connexins	Connexins show posttranscriptional modulations through glycosylation but also through phosphorylation, proteolysis, acetylation, nitrosylation, ubiquitination, lipidation, hydroxylation, methylation, and deamidation.	[52]
Connexin 43	Connexin 43 is expressed by endothelium and is involved in regulation of permeability. Glycosylation of connexin 43 is important for the regulation of their biological functions; inhibition of glycosylation enhances both basal and cAMP induced junctional communication.	[53–55]

*Adherent junctions*		
E-Cadherin	Modification of N-linked glycanes can affect their adhesive functions; glycosyltransferases are involved in this modulation, including acetylglucosaminyltransferases.	[56, 57]

*Transcellular transport*		
Caveolin-CD147	CD147 is an interaction partner of Caveolin-1; a significant biochemical property of CD147 is its high level of glycosylation. Glycosylation is important for its biological functions and glycosyltransferases involved in the biosynthesis of CD147 N-glycanes. Glycosylated extracellular matrix metalloproteinase inducer specifically associates with caveolin-1.	[58–60]
