# Supplementary material for: Metabolic Serum Profiles for Patients Receiving Allogeneic Stem Cell Transplantation: The Pretransplant Profile Differs for Patients with and without Posttransplant Capillary Leak Syndrome
Source: Dis Markers. 2015 Nov 2;2015:943430. doi: 10.1155/2015/943430 (PMC4644835; doi:10.1155/2015/943430)
Supplement: Supplementary file 1 — The description of the Supplementary Material should read: Supplementary Table 1. Classification of all metabolies analysed in the study, the pathways showing pretransplant alterations in patients with posttransplant capillary leak syndrome are marked with yellow. The marked pathways represent an extended list compared with the pathways presented in Figure 3; the ratio from the bioinformatical analysis is given for each Sub Pathway in parenthesis. [file 943430.f1.pdf]

Supplementary Table 1. Classification of all metabolies analysed in the study, the pathways showing pretransplant alterations in patients with posttransplant capillary leak syndrome are marked with yellow. The marked pathways represent an extended list compared with the pathways presented in Figure 3; the ratio from the bioinformatical analysis is given for each Sub Pathway in parenthesis.

Super Pathway	Sub Pathway	Biochemical Name	KEGG	PUBCHEM	HMDB
Amino Acid	Glycine, Serine and Threonine Metabolism	glycine	C00037	750	HMDB00123
		N-acetylglycine		10972	HMDB00532
		dimethylglycine	C01026	673	HMDB00092
		betaine	C00719	247	HMDB00043
		serine	C00065	5951	HMDB00187
		N-acetylserine		65249	HMDB02931
		threonine	C00188	6288	HMDB00167
		N-acetylthreonine	C01118	152204	
		O-acetylhomoserine	C01077	439389	
	Alanine and Aspartate Metabolism	alanine	C00041	5950	HMDB00161
		N-acetylalanine	C02847	88064	HMDB00766
		aspartate	C00049	5960	HMDB00191
		asparagine	C00152	6267	HMDB00168
		N-acetylaspartate (NAA)	C01042	65065	HMDB00812
	Glutamate Metabolism	glutamate	C00025	611	HMDB00148
		glutamine	C00064	5961	HMDB00641
		N-acetylglutamate	C00624	70914	HMDB01138
		N-acetylglutamine	C02716	182230	HMDB06029
		N-acetyl-aspartyl-glutamate (NAAG)	C12270	5255	HMDB01067
		pyroglutamine*		134508	
	Histidine Metabolism	histidine	C00135	6274	HMDB00177
		N-acetylhistidine	C02997	75619	HMDB32055
		1-methylhistidine	C01152	92105	HMDB00001
		3-methylhistidine	C01152	64969	HMDB00479
		N-acetyl-3-methylhistidine*		193270	
		N-acetyl-1-methylhistidine*			
		hydantoin-5-propionic acid	C05565	782	HMDB01212

		trans-urocanate	C00785	736715	HMDB00301
		imidazole propionate		70630	HMDB02271
		imidazole lactate	C05568	440129	HMDB02320
		1-methylimidazoleacetate	C05828	75810	HMDB02820
		4-imidazoleacetate	C02835	96215	HMDB02024
	Lysine Metabolism	lysine	C00047	5962	HMDB00182
		N2-acetyllysine	C12989	92907	HMDB00446
		N6-acetyllysine	C02727	92832	HMDB00206
		N-6-trimethyllysine	C03793	440120	HMDB01325
		2-aminoadipate	C00956	469	HMDB00510
		glutarate (pentanedioate)	C00489	743	HMDB00661
		glutarylcarnitine (C5)		71464488	HMDB13130
		3-methylglutarylcarnitine (1)		128145	HMDB00552
		3-methylglutarylcarnitine (2)		128145	HMDB00552
		pipecolate	C00408	849	HMDB00070
	Phenylalanine and Tyrosine Metabolism (1.96)	phenylalanine	C00079	6140	HMDB00159
		N-acetylphenylalanine	C03519	74839	HMDB00512
		phenylpyruvate	C00166	997	HMDB00205
		phenyllactate (PLA)	C05607	3848	HMDB00779
		phenylacetate	C07086	999	HMDB00209
		4-hydroxyphenylacetate	C00642	127	HMDB00020
		phenylacetylglutamine	C04148	92258	HMDB06344
		tyrosine	C00082	6057	HMDB00158
		N-acetyltyrosine		68310	HMDB00866
		4-hydroxyphenylpyruvate	C01179	979	HMDB00707
		3-(4-hydroxyphenyl)lactate	C03672	9378	HMDB00755
		phenol sulfate	C02180	74426	HMDB60015
		p-cresol sulfate	C01468	4615423	HMDB11635
		o-cresol sulfate		11615528	
		vanillylmandelate (VMA)	C05584	1245	HMDB00291
		3-methoxytyrosine		1670	HMDB01434
		3-methoxytyramine sulfate			
		homovanillate (HVA)	C05582	1738	HMDB00118

		gentisate	C00628	3469	HMDB00152
		3-[3-(sulfooxy)phenyl]propanoic acid		187488	
		3-(3-hydroxyphenyl)propionate	C11457	91	HMDB00375
		3-(4-hydroxyphenyl)propionate	C01744	10394	HMDB02199
		3-phenylpropionate (hydrocinnamate)	C05629	107	HMDB00764
		thyroxine	C01829	5819	HMDB01918
		5-hydroxymethyl-2-furoic acid	C20448	80642	HMDB02432
		2-hydroxyphenylacetate	C05852	11970	HMDB00669
	Tryptophan Metabolism (3.00)	tryptophan	C00078	6305	HMDB00929
		N-acetyltryptophan	C03137	700653	HMDB13713
		indolelactate	C02043	92904	HMDB00671
		indoleacetate	C00954	802	HMDB00197
		indolepropionate		3744	HMDB02302
		3-indoxyl sulfate		10258	HMDB00682
		kynurenine	C00328	161166	HMDB00684
		kynurenate	C01717	3845	HMDB00715
		3-hydroxykynurenine	C02794	89	HMDB00732
		xanthurenate	C02470	5699	HMDB00881
		5-hydroxyindoleacetate	C05635	1826	HMDB00763
		serotonin (5HT)	C00780	5202	HMDB00259
		indolebutyrate	C11284	8617	HMDB02096
		indoleacetylglutamine		25200879	HMDB13240
		tryptophan betaine	C09213	442106	HMDB61115
		C-glycosyltryptophan*			
		indole-3-carboxylic acid	C19837	69867	HMDB03320
	Leucine, Isoleucine and Valine Metabolism	leucine	C00123	6106	HMDB00687
		N-acetylleucine	C02710	70912	HMDB11756
		4-methyl-2-oxopentanoate	C00233	70	HMDB00695
		isovalerate	C08262	10430	HMDB00718

		isovalerylglycine		546304	HMDB00678
		isovalerylcarnitine		6426851	HMDB00688
		3-methylcrotonylglycine		169485	HMDB00459
		beta-hydroxyisovalerate		69362	HMDB00754
		beta-hydroxyisovaleroylcarnitine			
		alpha-hydroxyisovaleroyl carnitine*			
		alpha-hydroxyisovalerate		99823	HMDB00407
		methylsuccinate		10349	HMDB01844
		isoleucine	C00407	6306	HMDB00172
		allo-isoleucine		6950182;99288	
		N-acetylisoleucine		2802421	
		3-methyl-2-oxovalerate	C00671	47	HMDB03736
		2-methylbutyrylcarnitine (C5)		6426901	HMDB00378
		2-methylbutyrylglycine		193872	HMDB00339
		tiglyl carnitine		22833596	HMDB02366
		tigloylglycine		6441567	HMDB00959
		2-hydroxy-3-methylvalerate		164623	HMDB00317
		3-hydroxy-2-ethylpropionate		188979	HMDB00396
		ethylmalonate		11756	HMDB00622
		valine	C00183	6287	HMDB00883
		N-acetylvaline		66789	HMDB11757
		3-methyl-2-oxobutyrate	C00141	49	HMDB00019
		isobutyrylcarnitine		168379	HMDB00736
		isobutyrylglycine		10855600	HMDB00730
		3-hydroxyisobutyrate	C06001	87	HMDB00336
		alpha-hydroxyisocaproate	C03264	83697	HMDB00746
	Methionine, Cysteine, SAM and Taurine Metabolism (6.38)	methionine	C00073	6137	HMDB00696
		N-acetylmethionine	C02712	448580	HMDB11745
		N-formylmethionine	C03145	439750	HMDB01015
		methionine sulfone		69961	
		methionine sulfoxide	C02989	158980	HMDB02005

		S-adenosylhomocysteine (SAH)	C00021	439155	HMDB00939
		alpha-ketobutyrate	C00109	58	HMDB00005
		2-aminobutyrate	C02261	439691	HMDB00650
		2-hydroxybutyrate (AHB)	C05984	440864	HMDB00008
		cysteine	C00097	5862	HMDB00574
		cystine	C00491	67678	HMDB00192
		S-methylcysteine		24417	HMDB02108
		cysteine s-sulfate	C05824	115015	HMDB00731
		hypotaurine	C00519	107812	HMDB00965
		taurine	C00245	1123	HMDB00251
		N-acetyltaurine		159864	
	Urea cycle; Arginine and Proline Metabolism (7.86)	arginine	C00062	232	HMDB00517
		urea	C00086	1176	HMDB00294
		ornithine	C00077	6262	HMDB03374
		proline	C00148	145742	HMDB00162
		citrulline	C00327	9750	HMDB00904
		homoarginine	C01924	9085	HMDB00670
		homocitrulline	C02427	65072	HMDB00679
		dimethylarginine (SDMA + ADMA)	C03626	123831	HMDB01539
		N-acetylarginine	C02562	67427	HMDB04620
		N-delta-acetylornithine		9920500	
		N-methyl proline		557	
		trans-4-hydroxyproline	C01157	5810	HMDB00725
		pro-hydroxy-pro		11673055	HMDB06695
	Creatine Metabolism	creatine	C00300	586	HMDB00064
		creatinine	C00791	588	HMDB00562
		guanidinoacetate	C00581	763	HMDB00128
	Polyamine Metabolism	acisoga		129397	
		5-methylthioadenosine (MTA)	C00170	439176	HMDB01173
		N-acetylputrescine	C02714	122356	HMDB02064
		4-acetamidobutanoate	C02946	18189	HMDB03681
	Guanidino and Acetamido	4-guanidinobutanoate	C01035	500	HMDB03464

	Metabolism	guanidinosuccinate	C03139	97856	HMDB03157
	Glutathione Metabolism	cysteine-glutathione disulfide		4247235	HMDB00656
		cys-gly, oxidized		333293	
		5-oxoproline	C01879	7405	HMDB00267
Peptide	Gamma-glutamyl Amino Acid	gamma-glutamylalanine		440103	
		gamma-glutamylglutamate	C05282	92865	HMDB11737
		gamma-glutamylglutamine	C05283	150914	HMDB11738
		gamma-glutamylglycine		165527	HMDB11667
		gamma-glutamylhistidine		7017195	
		gamma-glutamylisoleucine*		14253342	HMDB11170
		gamma-glutamylleucine		151023	HMDB11171
		gamma-glutamyllysine		65254;14284565	HMDB03869
		gamma-glutamylmethionine		7009567	
		gamma-glutamylphenylalanine		111299	HMDB00594
		gamma-glutamylthreonine*			
		gamma-glutamyltryptophan		3989307	
		gamma-glutamyltyrosine		94340	HMDB11741
		gamma-glutamylvaline		7015683	HMDB11172
		gamma-glutamyl-2-aminobutyrate			
	Dipeptide Derivative	N-acetylcarnosine		9903482	HMDB12881
	Dipeptide	alanylleucine		259583	
		alanylphenylalanine		2080	
		alpha-glutamylglutamate	C01425	439500	HMDB28818
		alpha-glutamyltyrosine		351830	
		asparagylvaline		7019993	
		aspartylleucine		332962	
		aspartylphenylalanine		93078	HMDB00706
		cyclo(gly-pro)		126154	
		cyclo(leu-pro)		7074739	
		cyclo(L-phe-D-pro)*		6992198	
		cyclo(L-phe-L-pro)	C11847	9837725;443440	

		glutamine-leucine			
		glycylglycine	C02037	11163	HMDB11733
		glycylisoleucine		88079	
		glycylleucine	C02155	92843	HMDB00759
		glycylphenylalanine		92953	HMDB28848
		glycylproline		3013625	HMDB00721
		glycyltryptophan		1551340;92181	
		glycyltyrosine		92829	HMDB28853
		glycylvaline		97417	HMDB28854
		histidylalanine		351667	
		histidylleucine		189008;6992010	
		histidylphenylalanine		4466133	
		histidyltryptophan		6539217	
		isoleucylalanine		5246009;5246010	
		isoleucylaspartate			
		isoleucylglutamine		7020102	
		isoleucylglycine		342532	
		isoleucylisoleucine		7010568	
		isoleucylleucine		11644431	
		isoleucylvaline		5246011;435949	
		leucylalanine		259321	
		leucylaspartate		3328705	
		leucylglutamate		5259589;5259590	
		leucylglycine		79070	
		leucylisoleucine		7010534	
		leucylleucine	C11332	76807	HMDB28933
		leucylphenylalanine		259325	
		leucylserine		3621685	
		lysylleucine		4682588	
		phenylalanylalanine		6993123;5488196	
		phenylalanylarginine		10268029	
		phenylalanylaspartate		335051	
		phenylalanylglutamate		4422358	

		phenylalanylglycine		98207	
		phenylalanylleucine		4078229	
		phenylalanylphenylalanine		6993090;6993089	
		phenylalanylserine		9859812	
		phenylalanyltryptophan			
		phenylalanylvaline		4096934	
		prolylglycine		7408076;6426709	
		prolylphenylalanine		5226097	
		prolylvaline		152307;6992339	
		pyroglutamylglutamine			
		pyroglutamylglycine		152981	
		pyroglutamylvaline		152416	
		serylalanine			
		serylleucine		7015695	
		seryltyrosine		4575735	
		threonylphenylalanine		4099799;4099798	
		tryptophylasparagine			
		tryptophylglutamate		3634442	
		tryptophylphenylalanine		4427709	
		tyrosylglutamate		7009628	
		tyrosylglutamine			
		tyrosylglycine		259323	
		valylarginine		4266827	
		valylglycine		136487	
		valylisoleucine		5246012;5246013	
		valylleucine		352039	
		cis-Cyclo[L-ala-L-Pro]		6428987	
	Polypeptide	bradykinin, des-arg(9)	C00306	105044	HMDB04246
		HWESASXX*			
		XHWESASXXR*			
	Fibrinogen Cleavage Peptide	ADSGEGDFXAEGGGVR*		16133137	
		DSGEGDFXAEGGGVR*			
Carbohydrate	Glycolysis, Gluconeogenesis, and	1,5-anhydroglucitol (1,5-AG)	C07326	64960	HMDB02712

	Pyruvate Metabolism	glucose	C00031	79025	HMDB00122
		pyruvate	C00022	1060	HMDB00243
		lactate	C00186	612	HMDB00190
		glycerate	C00258	752	HMDB00139
	Pentose Metabolism	ribulose	C00309	151261	HMDB00621
		ribose	C00121	5779	HMDB00283
		ribitol	C00474	6912	HMDB00508
		xylonate	C05411	6602431	HMDB60256
		xylose	C00181	135191	HMDB00098
		xylitol	C00379	6912	HMDB02917
		arabinose	C00216	66308	HMDB00646
		threitol	C16884	169019	HMDB04136
		arabitol	C01904	94154	HMDB01851
		fucose	C01018	19466	HMDB00174
	Disaccharides and Oligosaccharides	sucrose	C00089	5988	HMDB00258
	Fructose, Mannose and Galactose Metabolism	fructose	C00095	5984	HMDB00660
		sorbitol	C00794	5780	HMDB00247
		mannose	C00159	18950	HMDB00169
		mannitol	C00392	6251	HMDB00765
		galactonate	C00880	128869	HMDB00565
	Aminosugar Metabolism (17.02)	glucuronate	C00191	444791	HMDB00127
		N-acetylneuraminate	C00270	439197	HMDB00230
		erythronate*		2781043	HMDB00613
Energy	TCA Cycle	citrate	C00158	311	HMDB00094
		aconitate [cis or trans] - group 51105	C00417,C02341		HMDB00072,HMDB00958
		alpha-ketoglutarate	C00026	51	HMDB00208
		succinylcarnitine			
		succinate	C00042	1110	HMDB00254
		fumarate	C00122	444972	HMDB00134
		malate	C00149	525	HMDB00156
	Oxidative Phosphorylation	acetylphosphate	C00227	186	HMDB01494

		phosphate	C00009	1061	HMDB01429
Lipid	Short Chain Fatty Acid	valerate	C00803	7991	HMDB00892
	Medium Chain Fatty Acid	caproate (6:0)	C01585	8892	HMDB00535
		caprylate (8:0)	C06423	379	HMDB00482
		caprate (10:0)	C01571	2969	HMDB00511
		10-undecenoate (11:1n1)			
		laurate (12:0)	C02679	3893	HMDB00638
		5-dodecenoate (12:1n7)		5312378	HMDB00529
	Long Chain Fatty Acid	myristate (14:0)	C06424	11005	HMDB00806
		myristoleate (14:1n5)	C08322	5281119	HMDB02000
		pentadecanoate (15:0)	C16537	13849	HMDB00826
		palmitate (16:0)	C00249	985	HMDB00220
		palmitoleate (16:1n7)	C08362	445638	HMDB03229
		margarate (17:0)		10465	HMDB02259
		10-heptadecenoate (17:1n7)		5312435	
		stearate (18:0)	C01530	5281	HMDB00827
		oleate (18:1n9)	C00712	445639	HMDB00207
		cis-vaccenate (18:1n7)	C08367	5282761	HMDB03231
		nonadecanoate (19:0)	C16535	12591	HMDB00772
		10-nonadecenoate (19:1n9)		5312513	HMDB13622
		arachidate (20:0)	C06425	10467	HMDB02212
		eicosenoate (20:1n9 or 11)		5282768	
		behenate (22:0)	C08281	8215	HMDB00944
		erucate (22:1n9)	C08316	5281116	HMDB02068
	Polyunsaturated Fatty Acid (n3 and n6)	stearidonate (18:4n3)	C16300	5312508	HMDB06547
		eicosapentaenoate (EPA; 20:5n3)	C06428	446284	HMDB01999
		docosapentaenoate (n3 DPA; 22:5n3)	C16513	6441454	HMDB01976
		docosahexaenoate (DHA; 22:6n3)	C06429	445580	HMDB02183
		docosatrienoate (22:3n3)	C16534	5312556	HMDB02823
		linoleate (18:2n6)	C01595	5280450	HMDB00673

		linolenate [alpha or gamma; (18:3n3 or 6)]	C06427	5280934	
		dihomo-linolenate (20:3n3 or n6)	C03242	5280581	HMDB02925
		arachidonate (20:4n6)	C00219	444899	HMDB01043
		adrenate (22:4n6)	C16527	5497181	HMDB02226
		docosapentaenoate (n6 DPA; 22:5n6)	C16513	6441454	HMDB13123
		docosadienoate (22:2n6)	C16533	5282807	
		dihomo-linoleate (20:2n6)	C16525	6439848	HMDB05060
		mead acid (20:3n9)		5312531	HMDB10378
	Fatty Acid, Branched	13-methylmyristic acid		151014	
		15-methylpalmitate (isobar with 2-methylpalmitate)		17903417	
		17-methylstearate		3083779	
	Fatty Acid, Dicarboxylate	2-hydroxyglutarate	C02630	43	HMDB00606
		maleate (cis-Butenedioate)	C01384	444266	HMDB00176
		pimelate (heptanedioate)	C02656	385	HMDB00857
		suberate (octanedioate)	C08278	10457	HMDB00893
		azelate (nonanedioate)	C08261	2266	HMDB00784
		sebacate (decanedioate)	C08277	5192	HMDB00792
		dodecanedioate	C02678	12736	HMDB00623
		tetradecanedioate		13185	HMDB00872
		hexadecanedioate	C19615	10459	HMDB00672
		octadecanedioate		70095	HMDB00782
		eicosanodioate		75502	
		3-carboxy-4-methyl-5-propyl-2-furanpropanoate (CMPF)		123979	HMDB61112
	Fatty Acid, Amide	palmitic amide		69421	
		oleamide	C19670	5283387	HMDB02117
	Fatty Acid, Amino	2-aminoheptanoate		227939	
		2-aminooctanoate		69522	HMDB00991
	Fatty Acid Synthesis	malonate (propanedioate)	C00383	867	HMDB00691
		2-methylmalonyl carnitine		53481628	HMDB13133

	Fatty Acid Metabolism (also BCAA Metabolism)	butyrylcarnitine	C02862	439829	HDMB02013
		butyrylglycine		88412	HMDB00808
		propionylcarnitine	C03017	107738	HMDB00824
		propionylglycine		98681	HMDB00783
		methylmalonate (MMA)	C02170	487	HMDB00202
	Fatty Acid Metabolism(Acyl Glycine)	hexanoylglycine		99463	HMDB00701
		N-octanoylglycine		84290	HMDB00832
		N-palmitoyl glycine		151008	
		N-linoleoylglycine		6433346	
	Fatty Acid Metabolism(Acyl Carnitine)	l	C02571	1	HMDB00201
		hydroxybutyrylcarnitine*		53481617	HMDB13127
		valerylcarnitine		6426903	HMDB13128
		hexanoylcarnitine		6426853	HMDB00705
		octanoylcarnitine	C02838	123701	HMDB00791
		decanoylcarnitine		10245190	HMDB00651
		cis-4-decenoyl carnitine			
		laurylcarnitine		10427569	HMDB02250
		myristoylcarnitine		53477791	HMDB05066
		palmitoylcarnitine	C02990	461	HMDB00222
		stearoylcarnitine		6426855	HMDB00848
		linoleoylcarnitine*		6450015	HMDB06469
		oleoylcarnitine		6441392;53477789	HMDB05065
		myristoleoylcarnitine*			
	Carnitine Metabolism	deoxycarnitine	C01181	134	HMDB01161
		carnitine	C00318	10917	HMDB00062
	Ketone Bodies	acetoacetate	C00164	96	HMDB00060
		3-hydroxybutyrate (BHBA)	C01089	441	HMDB00357
	Fatty Acid, Monohydroxy	alpha-hydroxycaproate		99824	HMDB01624
		2-hydroxyoctanoate		94180	HMDB02264
		2-hydroxydecanoate		21488	
		2-hydroxypalmitate		92836	HMDB31057
		2-hydroxystearate	C03045	69417	
		3-hydroxypropanoate	C01013	68152	HMDB00700

		3-hydroxyoctanoate		26613	HMDB01954
		3-hydroxydecanoate		26612	HMDB02203
		3-hydroxysebacate		3017884	HMDB00350
		3-hydroxylaurate		94216	HMDB00387
		5-hydroxyhexanoate		170748	HMDB00525
		16-hydroxypalmitate	C18218	10466	HMDB06294
		13-HODE + 9-HODE		43013	
	Fatty Acid, Dihydroxy	12,13-DiHOME	C14829	10236635	HMDB04705
		9,10-DiHOME	C14828	9966640	HMDB04704
		19,20-DiHDPA		16061148	
	Eicosanoid (8.51)	leukotriene B4	C02165	5280492	HMDB01085
		thromboxane B2	C05963	5283137	HMDB03252
		5-HETE	C04805	5280733	HMDB11134
		9-HETE		5312978	HMDB10222
		12-HETE		5312983	HMDB06111
		leukotriene B5		5283125	HMDB05073
	Endocannabinoid	oleic ethanolamide		5283454	HMDB02088
		palmitoyl ethanolamide	C16512	4671	HMDB02100
		N-oleoyltaurine		6437033	
		N-stearoyltaurine		168274	
		N-palmitoyltaurine			
	Inositol Metabolism	myo-inositol	C00137	892	HMDB00211
		chiro-inositol	C19891		HMDB34220
		scyllo-inositol	C06153	892	HMDB06088
		inositol 1-phosphate (I1P)	C04006	440194	HMDB00213
	Phospholipid Metabolism	choline	C00114	305	HMDB00097
		choline phosphate	C00588	1014	HMDB01565
		glycerophosphorylcholine (GPC)	C00670	71920	HMDB00086
		glycerophosphoethanolamine	C01233	123874	HMDB00114
		trimethylamine N-oxide	C01104	1145	HMDB00925

	Lysolipid	1-myristoylglycerophosphocholine (14:0)	C04230	460604	HMDB10379
		2-myristoylglycerophosphocholine*			
		1-myristoleoylglycerophosphocholine (14:1) *			
		1-pentadecanoylglycerophosphocholine (15:0)*			
		1-palmitoylglycerophosphocholine (16:0)		86554	
		2-palmitoylglycerophosphocholine		15061532	
		1-palmitoleoylglycerophosphocholine (16:1)*		24779461	
		2-palmitoleoylglycerophosphocholine*			
		1-margaroylglycerophosphocholine (17:0)	C04230	24779463	HMDB12108
		2-margaroylglycerophosphocholine*			
		1-stearoylglycerophosphocholine (18:0)		497299	
		2-stearoylglycerophosphocholine*		10208382	

		1-oleoylglycerophosphocholine (18:1)		16081932	
		2-oleoylglycerophosphocholine			
		1-linoleoylglycerophosphocholine (18:2n6)	C04100	11988421	
		2-linoleoylglycerophosphocholine*			
		1-linolenoylglycerophosphocholine (18:3n3)*			
		2-linolenoylglycerophosphocholine(18:3n3)*			
		1-nonadecanoylglycerophosphocholine(19:0)			
		1-dihomo-linoleoylglycerophosphocholine (20:2n6)*			
		1-arachidoylglycerophosphocholine (20:0)	C04230	24779473	HMDB10390
		1-eicosenoylglycerophosphocholine (20:1n9)*			
		2-eicosenoylglycerophosphocholine(20:1n9)*			
		1-eicosatrienoylglycerophosphocholine (20:3)*			
		2-eicosatrienoylglycerophosphocholine*			

		1-arachidonoylglycerophosphocholine (20:4n6)*	C05208		
		2-arachidonoylglycerophosphocholine*			
		1-eicosapentaenoylglycerophosphocholine (20:5n3)*			
		1-docosapentaenoylglycerophosphocholine (22:5n3)*			
		2-docosapentaenoylglycerophosphocholine (22:5n3)*			
		1-docosapentaenoylglycerophosphocholine (22:5n6)*			
		1-docosahexaenoylglycerophosphocholine (22:6n3)*			
		2-docosahexaenoylglycerophosphocholine*			
		1-palmitoylplasmenylethanolamine*			
		1-stearoylplasmenylethanolamine*			
		1-oleoylplasmenylethanolamine*			
		1-palmitoylglycerophosphoethanolamine		9547069	HMDB11503

		2-palmitoylglycerophosphoethanolamine*			
		1-margaroylglycerophosphoethanolamine*			
		1-stearoylglycerophosphoethanolamine		9547068	HMDB11130
		2-stearoylglycerophosphoethanolamine*			
		1-oleoylglycerophosphoethanolamine		9547071	HMDB11506
		2-oleoylglycerophosphoethanolamine*			
		1-palmitoleoylglycerophosphoethanolamine*			
		1-linoleoylglycerophosphoethanolamine*		52925130	HMDB11507
		2-linoleoylglycerophosphoethanolamine*			
		1-arachidonoylglycerophosphoethanolamine*		42607465	HMDB11517
		2-arachidonoylglycerophosphoethanolamine*			
		2-docosahexaenoylglycerophosphoethanolamine*			

		1-eicosatrienoylglycerophosphoethanolamine*			
		1-eicosapentaenoylglycerophosphoethanolamine*			
		1-docosahexaenoylglycerophosphoethanolamine*			
		1-palmitoylglycerophosphoinositol*			
		1-palmitoleoylglycerophosphoinositol*			
		1-stearoylglycerophosphoinositol			
		2-stearoylglycerophosphoinositol*			
		1-oleoylglycerophosphoinositol*			
		1-linoleoylglycerophosphoinositol*			
		2-linoleoylglycerophosphoinositol*			
		1-arachidonoylglycerophosphoinositol*			
		2-arachidonoylglycerophosphoinositol*			

		1-stearoylglycerophosphoserine*		9547101	
		1-palmitoylglycerophosphate	C04036	6419701	HMDB00327
		1-arachidonoylglyercophosphate			
		1-oleoylglycerophosphate		5497152	
		1-palmitoylglycerophosphoglycerol*		3300276	
		1-stearoylglycerophosphoglycerol			
		1-oleoylglycerophosphoglycerol*			
		2-nonadecanoylglycerophosphocholine(19:0)*			
		palmitoyl-arachidonoyl-glycerophosphocholine (1)*			
		palmitoyl-arachidonoyl-glycerophosphocholine (2)*			
		palmitoyl-linoleoyl-glycerophosphocholine (1)*			
		palmitoyl-linoleoyl-glycerophosphocholine (2)*			
		palmitoyl-oleoyl-glycerophosphocholine (1)*			
		stearoyl-arachidonoyl-glycerophosphoinositol (1)*			
		stearoyl-linoleoyl-glycerophosphocholine (1)*			
		stearoyl-linoleoyl-glycerophosphocholine (2)*			
	Glycerolipid Metabolism	glycerol	C00116	753	HMDB00131

		glycerol 3-phosphate (G3P)	C00093	754	HMDB00126
	Monoacylglycerol	1-myristoylglycerol (1-monomyristin)	C01885	79050	HMDB11561
		2-myristoylglycerol (2-monomyristin)		137938	
		1-pentadecanoylglycerol (1-monopentadecanoin)		190750	
		1-palmitoylglycerol (1-monopalmitin)		14900	HMDB31074
		2-palmitoylglycerol (2-monopalmitin)		123409	HMDB11533
		1-stearoylglycerol (1-monostearin)	D01947	24699	HMDB31075
		2-stearoylglycerol (2-monostearin)		79075	
		1-oleoylglycerol (1-monoolein)		5283468	HMDB11567
		2-oleoylglycerol (2-monoolein)		5319879	
		1-linoleoylglycerol (1-monolinolein)		5283469	
		2-linoleoylglycerol (2-monolinolein)		5365676	HMDB11538
		1-arachidonylglycerol	C13857	5282281	HMDB11572
		2-arachidonoyl glycerol	C13856	5282280	HMDB04666
		1-docosahexaenoylglycerol (1-monodocosahexaenoin)			
		1-dihomo-linolenylglycerol (alpha, gamma)			
	Sphingolipid Metabolism	sphinganine	C00836	3126	HMDB00269
		palmitoyl sphingomyelin		9939941	
		stearoyl sphingomyelin	C00550	6453725	HMDB01348
		oleoyl sphingomyelin		6443882	
		sphingosine 1-phosphate	C06124	5283560	HMDB00277
		sphingosine	C00319	5353955	HMDB00252
		myristoyl sphingomyelin*		11433862	

		myristoleoyl sphingomyelin*			
		nervonoyl sphingomyelin*			
		palmitoleoyl sphingomyelin*			
	Mevalonate Metabolism	3-hydroxy-3-methylglutarate	C03761	1662	HMDB00355
	Sterol	lathosterol	C01189	65728	HMDB01170
		cholesterol	C00187	11025495	HMDB00067
		7-alpha-hydroxy-3-oxo-4-cholestenoate (7-Hoca)	C17337	3081085	HMDB12458
		beta-sitosterol	C01753	222284	HMDB00852
		campesterol	C01789	173183	HMDB02869
	Steroid	pregnenolone sulfate		105074	HMDB00774
		21-hydroxypregnenolone monosulfate (1)		174681	
		21-hydroxypregnenolone monosulfate (2)		10670474	
		21-hydroxypregnenolone disulfate	C05485	134595	HMDB04026
		5alpha-pregnan-3alpha-ol,20-one sulfate			
		5alpha-pregnan-3beta,20beta-diol monosulfate (1)			
		5alpha-pregnan-3beta,20alpha-diol monosulfate (2)			
		5alpha-pregnan-3beta,20alpha-diol disulfate			
		5alpha-pregnan-3(alpha or beta),20beta-diol disulfate		5127902	
		pregnen-diol disulfate*			
		pregn steroid monosulfate*			
		pregnanediol-3-glucuronide		123796	
		cortisol	C00735	5754	HMDB00063
		corticosterone	C02140	5753	HMDB01547
		11-dehydrocorticosterone			
		cortisone	C00762	222786	HMDB02802

		dehydroisoandrosterone sulfate (DHEA-S)	C04555	12594	HMDB01032
		16a-hydroxy DHEA 3-sulfate			
		epiandrosterone sulfate	C07635		HMDB00365
		androsterone sulfate		159663	HMDB02759
		4-androsten-3beta,17beta-diol monosulfate (1)			HMDB03818
		4-androsten-3beta,17beta-diol monosulfate (2)			
		4-androsten-3alpha,17alpha-diol monosulfate (2)			
		4-androsten-3alpha,17alpha-diol monosulfate (3)			
		4-androsten-3beta,17beta-diol disulfate (1)	C04295	10634	HMDB03818
		4-androsten-3beta,17beta-diol disulfate (2)	C04295	10634	HMDB03818
		5alpha-androstan-3beta,17beta-diol monosulfate (1)			
		5alpha-androstan-3alpha,17beta-diol monosulfate (1)			
		5alpha-androstan-3alpha,17alpha-diol monosulfate			
		5alpha-androstan-3beta,17beta-diol monosulfate (2)			
		5alpha-androstan-3alpha,17alpha-diol disulfate			
		5alpha-androstan-3beta,17alpha-diol disulfate			
		5alpha-androstan-3alpha,17beta-diol disulfate			
		5alpha-androstan-3beta,17beta-diol disulfate	C12525	242332	HMDB00493

		andro steroid monosulfate (1)*	C04555		HMDB02759
		estrone 3-sulfate	C02538	3001028	HMDB01425
		testosterone sulfate	C00535	119207	HMDB02833
		11-ketoetiocholanolone glucuronide			
		etiocholanolone glucuronide			
		5alpha-androstan-3alpha,17beta-diol monosulfate (2)			
		17alpha-hydroxypregnanolone glucuronide			
		5alpha-pregnan-3alpha,20beta-diol monosulfate (4)			
	Primary Bile Acid Metabolism	cholate	C00695	221493	HMDB00619
		glycocholate	C01921	10140	HMDB00138
		taurocholate	C05122	6675	HMDB00036
		chenodeoxycholate	C02528	10133	HMDB00518
		glycochenodeoxycholate	C05466	12544	HMDB00637
		taurochenodeoxycholate	C05465	387316	HMDB00951
		tauro-beta-muricholate		168408	HMDB00932
	Secondary Bile Acid Metabolism	deoxycholate	C04483	222528	HMDB00626
		glycodeoxycholate	C05464	3035026	HMDB00631
		taurodeoxycholate	C05463	2733768	HMDB00896
		glycolithocholate	C15557	115245	HMDB00698
		glycolithocholate sulfate*	C11301	72222	HMDB02639
		taurolithocholate	C02592	10595	HMDB00722
		taurolithocholate 3-sulfate	C03642	440071	HMDB02580
		ursodeoxycholate	C07880	31401	HMDB00946
		glycoursodeoxycholate		12310288	HMDB00708
		tauroursodeoxycholate		9848818	HMDB00874
		7-ketolithocholate		444262	HMDB00467

		glycohyocholate			
		hyodeoxycholate	C15517	5283820	HMDB00733
		glycocholenate sulfate*			
		taurocholenate sulfate			
		7-ketodeoxycholate		188292	HMDB00391
		3beta,7alpha-dihydroxy-5-cholestenoate			
		3b-hydroxy-5-cholenoic acid		92997	HMDB00308
Nucleotide	Purine Metabolism, (Hypo)Xanthine/Inosine containing	inosine	C00294	6021	HMDB00195
		hypoxanthine	C00262	790	HMDB00157
		xanthine	C00385	1188	HMDB00292
		xanthosine	C01762	64959	HMDB00299
		urate	C00366	1175	HMDB00289
		allantoin	C02350	204	HMDB00462
	Purine Metabolism, Adenine containing (7.30)	adenosine 3',5'-cyclic monophosphate (cAMP)	C00575	6076	HMDB00058
		adenosine	C00212	60961	HMDB00050
		adenine	C00147	190	HMDB00034
		N1-methyladenosine	C02494	27476	HMDB03331
		N6-methyladenosine		102175	HMDB04044
		N6-carbamoylthreonyladenosine		161466	HMDB41623
		N6-succinyladenosine			HMDB00912
	Purine Metabolism, Guanine containing (17.02)	guanosine	C00387	6802	HMDB00133
		7-methylguanine	C02242	11361	HMDB00897
		2'-O-methylguanosine	C04545		
		N1-methylguanosine		96373	HMDB01563
		N2-methylguanosine		3035422	HMDB05862
		N2,N2-dimethylguanosine		92919	HMDB04824
	Pyrimidine Metabolism, Orotate containing	dihydroorotate	C00337	648	HMDB03349
		orotate	C00295	967	HMDB00226
		orotidine	C01103	92751	HMDB00788
	Pyrimidine Metabolism, Uracil	uridine	C00299	6029	HMDB00296

	containing	uracil	C00106	1174	HMDB00300
		pseudouridine	C02067	15047	HMDB00767
		5-methyluridine (ribothymidine)		445408	HMDB00884
		3'-O-Methyluridine			
		5,6-dihydrouracil	C00429	649	HMDB00076
		2'-deoxyuridine	C00526	13712	HMDB00012
		3-ureidopropionate	C02642	111	HMDB00026
		beta-alanine	C00099	239	HMDB00056
		N-acetyl-beta-alanine	C01073	76406	
	Pyrimidine Metabolism, Cytidine containing (25.53)	cytidine	C00475	6175	HMDB00089
		N4-acetylcytidine		107461	HMDB05923
	Pyrimidine Metabolism, Thymine containing	5,6-dihydrothymine	C00906	93556	HMDB00079
	Purine and Pyrimidine Metabolism	methylphosphate		13130	
Cofactors and Vitamins	Nicotinate and Nicotinamide Metabolism	quinolinate	C03722	1066	HMDB00232
		nicotinamide	C00153	936	HMDB01406
		1-methylnicotinamide	C02918	10129985	HMDB00699
		trigonelline (N'-methylnicotinate)	C01004	5570	HMDB00875
		N1-Methyl-2-pyridone-5-carboxamide	C05842	69698	HMDB04193
	Riboflavin Metabolism	riboflavin (Vitamin B2)	C00255	493570	HMDB00244
	Pantothenate and CoA Metabolism	pantothenate	C00864	6613	HMDB00210
	Ascorbate and Aldarate Metabolism	ascorbate (Vitamin C)	C00072		HMDB00044
		threonate	C01620	151152	HMDB00943
		arabonate	C00878	122045	HMDB00539
		oxalate (ethanedioate)	C00209	971	HMDB02329
		gulonic acid*		9794176	
	Tocopherol Metabolism	alpha-tocopherol	C02477	14985	HMDB01893
		beta-tocopherol	C14152	6857447	HMDB06335
		gamma-tocopherol	C02483	14986	HMDB01492

		gamma-CEHC		133098	HMDB01931
		gamma-CEHC glucuronide*			
		alpha-CEHC glucuronide*			
		alpha-CEHC sulfate			
	Tetrahydrobiopterin Metabolism	biopterin	C06313	445040	HMDB00468
	Hemoglobin and Porphyrin Metabolism	bilirubin (Z,Z)	C00486	5280352	HMDB00054
		bilirubin (E,E)*		5315454	
		bilirubin (E,Z or Z,E)*		5799469	
		biliverdin	C00500	5353439	HMDB01008
		I-urobilinogen	C05790	26818	HMDB04157
		L-urobilin	C05793	5280818	HMDB04159
	Vitamin B6 Metabolism	pyridoxate	C00847	6723	HMDB00017
Xenobiotics	Benzoate Metabolism	hippurate	C01586	464	HMDB00714
		2-hydroxyhippurate (salicylurate)	C07588	10253	HMDB00840
		3-hydroxyhippurate		450268	HMDB06116
		4-hydroxyhippurate		151012	HMDB13678
		benzoate	C00180	243	HMDB01870
		4-hydroxybenzoate	C00156	135	HMDB00500
		catechol sulfate	C00090	3083879	HMDB59724
		O-methylcatechol sulfate		22473	
		3-methyl catechol sulfate (1)			
		3-methyl catechol sulfate (2)			
		4-methylcatechol sulfate			
		methyl-4-hydroxybenzoate	D01400	7456	HMDB32572
		2-ethylphenylsulfate			
		3-ethylphenylsulfate	C14386		
		4-ethylphenylsulfate	C13637		
		4-vinylphenol sulfate	C05627	6426766	HMDB04072
	Xanthine Metabolism	caffeine	C07481	2519	HMDB01847
		paraxanthine	C13747	4687	HMDB01860
		theobromine	C07480	5429	HMDB02825

		theophylline	C07130	2153	HMDB01889
		1-methylurate	C16359	69726	HMDB03099
		7-methylurate		69160	
		1,3-dimethylurate		70346	HMDB01857
		1,7-dimethylurate	C16356	91611	HMDB11103
		3,7-dimethylurate	C16360	83126	HMDB01982
		1,3,7-trimethylurate	C16361	79437	HMDB02123
		1-methylxanthine	C16358	80220	HMDB10738
		3-methylxanthine	C16357	70639	HMDB01886
		7-methylxanthine	C16353	68374	HMDB01991
		5-acetylamino-6-amino-3-methyluracil	C16366	88299	HMDB04400
		5-acetylamino-6-formylamino-3-methyluracil	C16365	108214	HMDB11105
	Tobacco Metabolite	cotinine		854019	HMDB01046
		hydroxycotinine		10219774	HMDB01390
		cotinine N-oxide		9815514	HMDB01411
	Food Component/Plant (1.7)	2-piperidinone		12665	
		sucralose	C12285	71485	HMDB31554
		levulinate (4-oxovalerate)		11579	HMDB00720
		1,6-anhydroglucose		2724705	HMDB00640
		2,3-dihydroxyisovalerate	C04039	677	HMDB12141
		2-isopropylmalate	C02504	77	HMDB00402
		2-oxindole-3-acetate		3080590	
		3-hydroxyindolin-2-one	C11130	6097	
		betonicine	C08269	164642	HMDB29412
		gluconate	C00257	10690	HMDB00625
		N-acetylalliin			
		cinnamoylglycine		709625	HMDB11621
		dihydroferulic acid		14340	
		ergothioneine	C05570	3032311	HMDB03045
		erythritol	C00503	222285	HMDB02994
		ferulate	C01494	445858	HMDB00954

		ferulic acid 4-sulfate		6305574	HMDB29200
		glycyrrhetinate	C02283	18526330	HMDB11628
		homostachydrine*	C08283	441447	HMDB33433
		methyl indole-3-acetate		74706	HMDB29738
		N-(2-furoyl)glycine		21863	HMDB00439
		piperine	C03882	638024	HMDB29377
		quinate	C00296	6508	HMDB03072
		saccharin	D01085	5143	HMDB29723
		stachydrine	C10172	115244	HMDB04827
		tartarate	C00898	444305	HMDB00956
		theanine	C01047	439378	HMDB34365
		thymol sulfate	C09908		HMDB01878
		4-allylphenol sulfate			
		methyl glucopyranoside (alpha + beta)			
	Bacterial/Fungal	tartronate (hydroxymalonate)	C02287	45	HMDB35227
	Drug	vancomycin		14969	
		2-hydroxyacetaminophen sulfate*			
		2-methoxyacetaminophen sulfate*			
		3-(cystein-S-yl)acetaminophen*		5233914	
		3-(N-acetyl-L-cystein-S-yl) acetaminophen		83967	
		4-acetaminophen sulfate	C06804	83939	HMDB59911
		4-acetamidophenol	C06804	1983	HMDB01859
		p-acetamidophenylglucuronide		83944	HMDB10316
		2-methoxyacetaminophen glucuronide*			
		salicyluric glucuronide*			
		ibuprofen acyl glucuronide		163959	
		ibuprofen	D00126	3672	HMDB01925

		2-hydroxyibuprofen			
		carboxyibuprofen		10444113	
		1-hydroxy-2-naphthalenecarboxylate	C03203	6844	
		4-acetylphenol sulfate		4684006	
		6-oxopiperidine-2-carboxylic acid		3014237	
		allopurinol		2094	
		allopurinol riboside			
		amphotericin B	C06573	5280965	HMDB14819
		codeine	C06174	5284371	HMDB04995
		O-desmethylvenlafaxine			
		doxycycline			
		escitalopram		146570	HMDB05028
		furosemide	D00331	3440	HMDB01933
		hydrochlorothiazide	C07041	3639	HMDB01928
		hydroquinone sulfate	C00530	161220	HMDB02434
		lidocaine	D00358	3676	HMDB14426
		metformin	C07151	4091	HMDB01921
		methotrexate	C01937	126941	HMDB14703
		metoprolol	D02358	4171	HMDB01932
		metoprolol acid metabolite*		62936	
		N-ethylglycinexylidide	C16561	24415	HMDB60656
		omeprazole	C07324	4594	HMDB01913
		oxypurinol	D02365	4644	HMDB00786
		pantoprazole	C11806	4679	HMDB05017
		pivaloylcarnitine		126894	HDMB41993
		ranitidine	D00422	3001055	HMDB01930
		salicylate	C00805	338	HMDB01895
		venlafaxine	C07187	5656	HMDB05016
		veratric acid		7121	
		warfarin			
		N,O-didesmethyl venlafaxine*		3451347	

		N,O-didesmethylvenlafaxine glucuronide*			
	Chemical (3.4)	1,2-propanediol	C00583	1030	HMDB01881
		2-pyrrolidinone		12025	HMDB02039
		sulfate*	C00059	1118	HMDB01448
		O-sulfo-L-tyrosine		514186	
		ethyl glucuronide		152226	
		2-aminophenol sulfate		181670	HMDB61116
		2-ethylhexanoate		8697	HMDB31230
		2-hydroxyisobutyrate		11671	HMDB00729
		S-(3-hydroxypropyl)mercapturic acid (HPMA)		3371179	
		dimethyl sulfone	C11142	6213	HMDB04983
		ectoine	C06231	126041	
		HEPES		23831	
		pepstatin A			
		phenylcarnitine*			
		N-methylpipecolate		11862129;11286529	
